# Breast tumor movements analysis using MRI scans in prone and supine positions

**DOI:** 10.1038/s41598-020-61802-9

**Published:** 2020-03-17

**Authors:** Chuan-Bing Wang, Sangwook Lee, Taehun Kim, Dayeong Hong, Guk Bae Kim, Ga Young Yoon, Hak Hee Kim, Namkug Kim, BeomSeok Ko

**Affiliations:** 1 Department of Radiology, First Affiliated Hospital of Nanjing Medical University, 300 Guangzhou Road, Nanjing, Jiangsu China; 2 Department of Radiology, University of Ulsan College of Medicine, Asan Medical Center, 388-1 Pungnap2-dong, Songpa-gu, Seoul South Korea; 3 Department of Convergence Medicine, University of Ulsan College of Medicine, Asan Medical Center, 388-1 Pungnap-dong, Songpa-gu, Seoul South Korea; 4 ANYMEDI Inc., 388-1 Pungnap-dong, Songpa-gu, Seoul South Korea; 5 Department of Breast Surgery, University of Ulsan College of Medicine, Asan Medical Center, 388-1 Pungnap-dong, Songpa-gu, Seoul South Korea

**Keywords:** Breast cancer, Breast cancer

## Abstract

We quantitatively evaluated breast tumor movement and volume changes between magnetic resonance imaging (MRI) scans in prone and supine positions. Twenty-seven breast tumor patients who received neoadjuvant systemic therapy (NST) for breast-conserving surgery were studied. Before and after NST, MRI scans in prone and supine positions were performed immediately. Tumor segmentation, volume, and position of tumors were evaluated in both positions. Average tumor volumes in prone and supine positions did not significantly differ (*p* = 0.877). Tumor movement from prone to supine positions from the origin of the bottom center of the sternum was strongly correlated with the distance from the tumor center to the chest wall (*r* = 0.669; *p* < 0.05). Tumor changes from prone to supine positions measured from the origin of the nipple depended on the location of the tumor in the breast. The prone-to-supine movement of all tumors from the origin of the bottom center of the sternum tended to move outward from the sagittal centerline of the body on the coronal plane, to the inside of the body on the sagittal plane, and outward and downward close to the body on the axial plane, which might help in planning operations using prone MRI in supine-position breast cancer surgery.

## Introduction

For breast cancer patients, breast-conserving surgery (BCS) has the advantage of improving cosmetic satisfaction and quality of life compared with mastectomy. Because the tumor-positive margin in BCS is highly correlated with the recurrence rate of breast cancer, it is important to have adequate margin resection. Therefore, the area of the tumor should be accurately predicted before surgery; magnetic resonance imaging (MRI) is well-known to be more accurate than mammography or ultrasonography (USG)^1–4^. MRI is a non-invasive imaging modality and is particularly useful for the scanning and detection of abnormalities in soft tissue structures in the body^5,6^. Waljee *et al*.^1^ reported that the sensitivity and specificity of MRI are high at 90% and 85%, respectively, in detecting breast tumors. However, several studies have demonstrated that MRI assessment before surgery fails to improve post-operative margin status and subsequent local recurrence, even compared with conventional imaging modalities^7,8^. Houssami *et al*.^9^ reported that a meta-analysis of pre-operative MRI and breast tumor recurrence also shows that pre-operative MRI for staging breast tumors does not reduce the risk of local or distant recurrence. A few existing studies indicated that the lack of a consistent strategy to manage MRI data led to an inappropriate interpretation of pre-operative breast MRI, which led to inaccurate surgical resection^2,3^. To improve the diagnostic accuracy of MRI, the scan is generally performed in the prone position with a breast coil, which could be quite different from the supine position during surgery. The MRI scan at the supine position is also difficult to perform due to low accuracy of tumor diagnosis due to motion artifacts and low contrast in MRI. Telegrafo *et al*.^4^ showed that a key issue for clinical use of pre-operative breast MRI is how to transfer the three-dimensional (3D) information obtained from the patient in prone position to the operating room, since the surgical treatment is performed in the supine position^4,10^. If the location of MRI-detected tumors in the supine position could be predicted, those predicted positions could be used as a surgical planning guide for the surgeon^11,12^, which could then promote a decrease in the re-excision rate of BCS.

The nipple plays a very important role in the positioning of the tumor as a natural marker in the operating room. One study reported that lesion-to-nipple distance and circumferential change from the nipple needs to be considered for lesion detection in MRI and ultrasound images^4^. However, ultrasound (US) has the obvious drawback of being unable to reveal subtle lesions^13^. Carbonaro *et al*.^14^ also suggested that lesion-to-nipple distance may be the most reliable measure used for second-look breast US, and that the median movement on the medial-sagittal plane of nipple distances in axial view was less than 1 cm when comparing breast MRIs in prone versus supine positions. Based on this study, the distance of the tumors at nipple origin was measured to determine the movement of the breast tumor in prone and supine positions. However, this distance may vary due to variation in the nipple position according to the amount of fatty tissue of the breast. To address this shortcoming, the distance of the tumors from the chest wall should be measured and compared to those at the nipple origin. The study by Satake *et al*.^11^ assessed the prediction of prone-to-supine tumor movement in the breast considering movement direction. However, only movement in the coronal plane was analyzed, which cannot show the movement of the tumor in three-dimensional (3D) coordinates.

Therefore, this study aims to evaluate the various kinds of spatial and volumetric changes including the distances from the lesion to the nipple origin and the origin of the bottom center of the sternum, and the distance of tumor origin to the chest wall, to predict more accurate tumor positions and volume changes for surgical planning in the operating room.

## Methods and Materials

### Patients

The study was approved by the institutional review board of Asan Medical Center (No. 2017–1341). The requirement of informed consent from patients was waived by the institutional review board. The imaging data were de-identified in accordance with the Health Insurance Portability and Accountability Act privacy rule. The study was conducted using a total of four types of MRI scans for 31 patients: (1) Pre-prone MRI, prone position before neoadjuvant systemic therapy (NST); (2) Pre-supine MRI, supine position before NST; (3) Post-prone MRI, prone position after NST; (4) Post-supine MRI, supine position after NST for BCS purposes between April 2016 and October 2017. Among them, the scans of four patients were excluded: one had received breast augmentation, and in the other three, the tumor margin of the pre- or post-supine MRIs was hard to find due to the outflow of the contrast agent. Thus, a total of 27 patients with 43 breast tumors were included in this study. Their age range was 36–65 years, and the mean age was 47.2 years. We chose the largest tumor in every case as the analysis target (15 left, 12 right; 26 invasive ductal carcinomas (IDC), 1 metaplastic carcinoma with osseous differentiation).

### MRI protocol

Breast imaging was performed with a 3.0 T MRI system (Ingrain; Philips Healthcare, Netherlands) with a bilateral dedicated four-element breast coil. Patients underwent a routine standard MRI protocol performed in prone position and then were repositioned in supine position. The standard breast MRI protocol included a T2 SPAIR (spectral attenuated inversion recovery), a T1 without fat suppression, a STIR (short-tau inversion recovery sequence), a DWI (diffusion-weighted MRI; b = 0,1000 s/mm^2^), and a dynamic perfusion study with an intravenous injection of 0.1 mmol/kg of gadopentetate dimeglumine (MultiHance, Gd-BOPTA; Bracco Imaging SpA, Milan, Italy), followed by a flush of 20 ml of saline solution at 2 ml/s. The dynamic study involved one pre-contrast acquisition followed by five post-contrast acquisitions of T1 weighted high resolution volume examinations (THRIVE; TR (repetition time) = 4.1 ms, TE (echo time) = 1.8 ms, slice thickness = 0.9 mm, pixel size = 0.9 × 0.9 mm). Immediately after the acquisition of the last dynamic series, the patient was extracted from the magnet, the breast coil was removed, and the patient was invited to assume the supine position. Thereafter, a thoracic four-channel surface coil was positioned over the breast surface. An mDixon (multi-point Dixon) sequence was used for acquiring MRI in the supine position with the following technical parameters: TR/TE = 4.9/0.0 ms, fractional anisotropy (FA) = 10°, voxel size = 0.75 × 0.75 × 2.0 mm, matrix = 320 × 320, and field of view (FOV) = 380 mm^14^.

### Tumor segmentation and measurement

**Figure 1:**
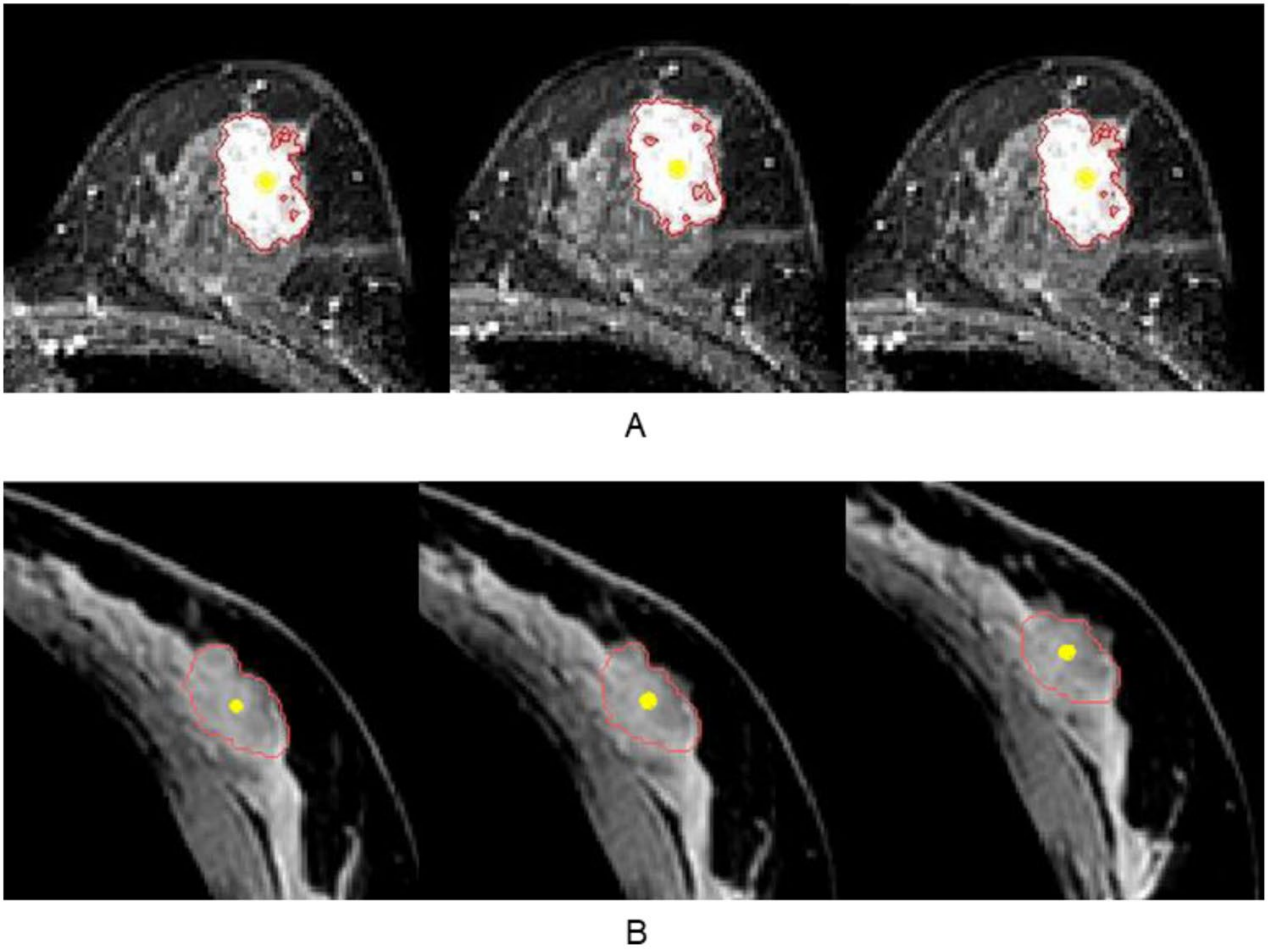
Tumor segmentation in a typical case with stage III invasive ductal carcinoma of the left breast. (**A**) Axial contrast-enhanced T1 weighted MRI was used for the prone position segmentation. (**B**) Axial mDIXON MRI was used for supine position segmentation (red line: tumor margin of segmentation mask, and yellow point: center point of tumor).

All MRI data were transferred and analyzed using Mimics Medical 17 (Materialise Inc, Belgium), which is software for imaging segmentation. All the tumor segmentation in prone and supine MRIs were performed by a breast MRI expert and confirmed by a breast surgeon. In prone position, contrast-enhanced T1 weighted MRI with clear tumor phase was selected for the segmentation. In supine position, mDixon MRI was used for these segmentations considering prone MRIs (Fig. 1). During segmentation, the tools of thresholding and region growing with cropped mask were used. At the same time, breast vessels, cysts, and other visible signs were used for references to confirm tumor margins, especially in supine MRI where the contrast agent might flow out and it was sometimes difficult to find the tumor margin. After segmentation, a 3D surface model of the tumor was generated and evaluated for tumor volume in both positions.

**Figure 2:**
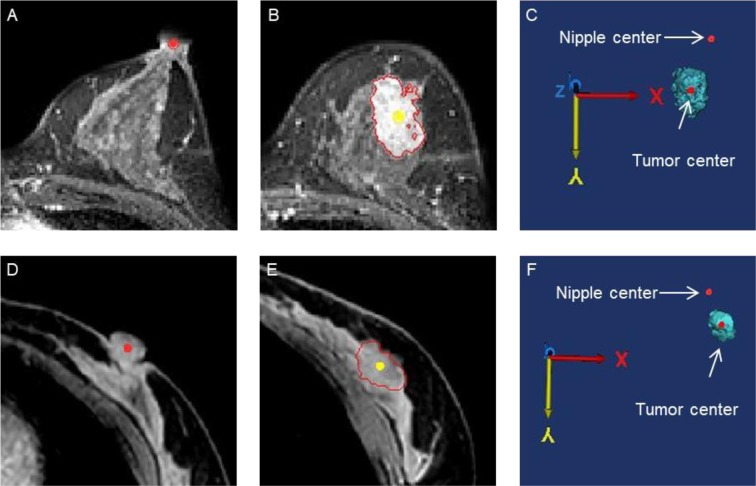
The distance from the nipple origin to the tumor origin in three-dimensional (3D) coordinates was visualized in the prone (**A–C**) and supine (**D–F**) positions. (**A**) The nipple origin was manually placed on the axial images (red point). (**B**) The tumor origin (yellow point) was evaluated through the center of gravity function. (**C**) The distance from the nipple origin to the tumor origin were evaluated in 3D coordinates.

**Figure 3:**
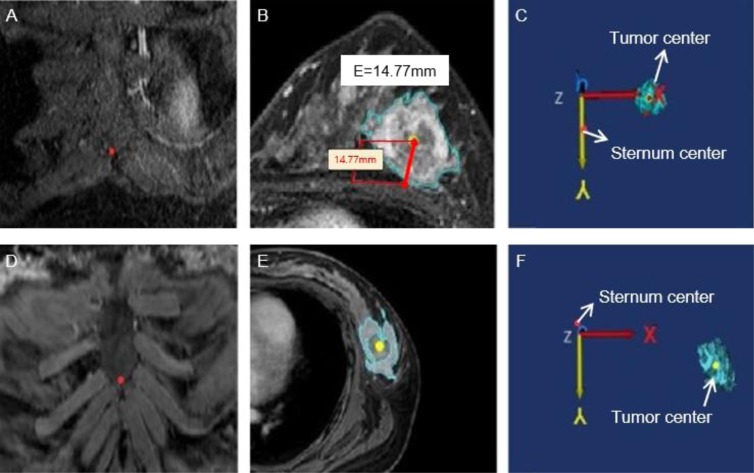
The origin of the bottom center of the sternum and the tumor center (red point) in 3D coordinates were obtained in the prone (**A–C**) and supine (**D–F**) positions. (**A**) The origin of the bottom center of the sternum (red point) was manually placed on coronal images. (**B**) The tumor center (yellow point) was evaluated through the center of gravity function and the distance from the tumor center to the chest wall was measured (red line). (**C**) The distance from the origin of the bottom center of the sternum to the tumor center were measured in 3D coordinates.

For each position, we obtained the center point of the tumor through the center of gravity function in Mimics (Fig. 2B,E). At the same time, the nipple origin (Fig. 2A,D), and the origin of the bottom center of the sternum (Fig. 3A,D) were manually placed in MRIs. Each point was measured in the X- (left-right), Y- (anterior-posterior), and Z- (superior-inferior) coordinates (Figs. 2C,F and 3C,F). To center the nipple origin as an origin $(0,\,0,\,0)$, the $({\rm{X}},\,Y,\,Z)$ coordinates of the nipple origin were subtracted from the (X, Y, Z) coordinates of the tumor origin for prone position as $({{\rm{X}}}_{a},{Y}_{b},{Z}_{c})$, and supine position as $({{\rm{X}}}_{a}^{\text{'}},\,{{\rm{Y}}}_{b}^{\text{'}},\,{{\rm{Z}}}_{c}^{\text{'}})$^14^.

The total distance of prone-to-supine tumor movement (total movement) (1) was calculated as follows.

1 $${\rm{D}}=\sqrt{{({{\rm{X}}}_{{\rm{a}}}-{{\rm{X}}}_{a}^{\text{'}})}^{2}+{({{\rm{Y}}}_{{\rm{a}}}-{{\rm{Y}}}_{a}^{\text{'}})}^{2}+{({{\rm{Z}}}_{{\rm{a}}}-{{\rm{Z}}}_{a}^{\text{'}})}^{2}}({\rm{mm}})$$

where $({{\rm{X}}}_{a},{Y}_{b},{Z}_{c})$ is the tumor center in prone position, and $({{\rm{X}}}_{a}^{\text{'}},\,{{\rm{Y}}}_{b}^{\text{'}},\,{{\rm{Z}}}_{c}^{\text{'}})$ is the tumor center in supine position.

If the origin of the bottom center of the sternum is regarded as an origin, total movement from prone to supine position and distance from tumor origin to the origin of the bottom center of the sternum were re-calculated in a similar manner. In addition, an orthogonal line from the tumor center to the chest wall was manually determined in prone MRI (Fig. 3B) and the length of the line was evaluated.

## Data Analysis and Statistics

Paired *t*-tests were performed to compare the tumor volumes in prone and supine positions and the total movements between the origin of the nipple and the origin of the bottom center of the sternum. Pearson’s correlation coefficients were also evaluated to analyze the associations among the tumor movements from prone to supine positions, the distance from the tumor center to the origin of the bottom center of the sternum, and the distance from the tumor center to the chest wall. Analyses were performed using the SPSS version 22 software (IBM, Chicago, IL, USA), with a *p*-value less than 0.05 considered as significant.

## Results

**Figure 4:**
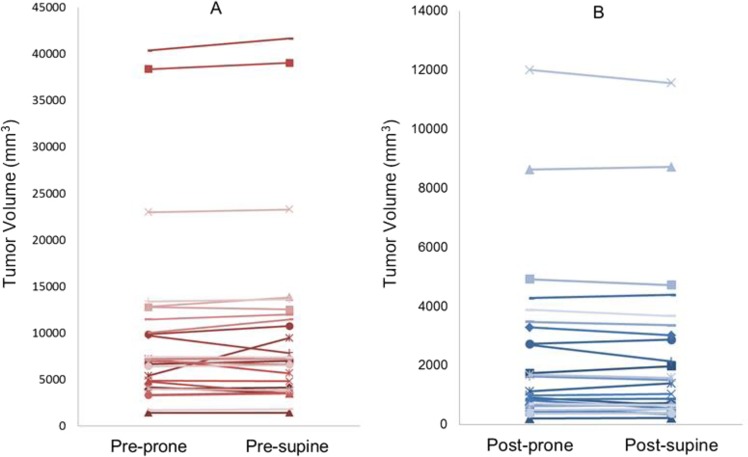
Tumor volume changes from prone to supine positions. (**A**) Pre-NST (**B**) Post-NST (NST: neoadjuvant systemic therapy).

**Figure 5:**
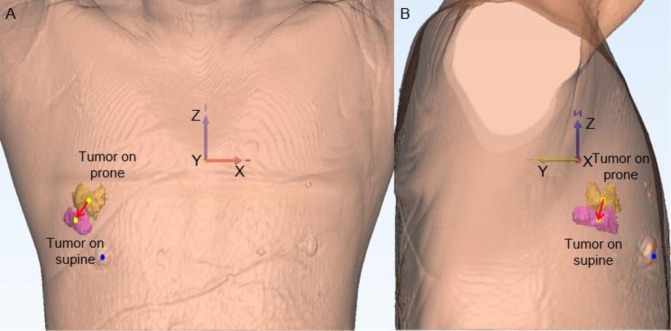
A case of tumor movement at the nipple origin from prone to supine positions. (**A**) and (**B**) Show that the tumor located in the outer area in the prone position moved outward and downward along the z-axis. (**A**) Presents a frontal view of the 3D body surface and the nipple origin (blue point) in supine position, and two tumors from the prone position (yellow) and supine position (pink). (**B**) Is a right-side view. (Yellow points: the origins of tumors; red arrow: tumor movement from prone to supine positions).

**Figure 6:**
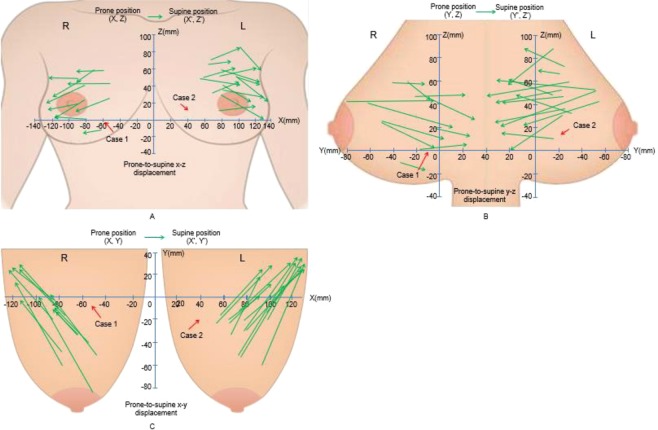
Coronal (X-Z), sagittal (Y-Z), and axial (X-Y) views of the bilateral breasts at the origin of the bottom center of the sternum (0, 0). The vectors of the tumors of 27 patients’ movements from the prone to supine positions on the coronal (**A**), sagittal (**B**), and axial (**C**) views were visualized. X- (left-right), Y- (anterior-posterior), and Z- (superior-inferior) coordinates (unit: mm).

**Figure 7:**
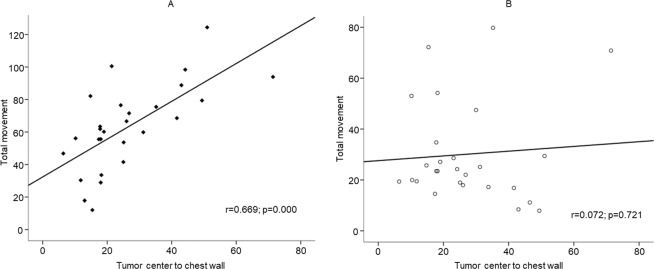
(**A**) Correlation between tumor movement at the origin of the bottom center of the sternum and the distance from the tumor center to the chest wall (r = 0.669; p < 0.05). (**B**) Correlation between tumor movement at the nipple origin and the distance from the tumor center to the chest wall (r = 0.072; p = 0.721).

The tumor volumes of prone and supine positions (mean ± SD) were 9,797.3 ± 9,390.6 mm^3^ and 9,795.5 ± 9,413.1 mm^3^, respectively, with no significant difference (*p* = 0.877) (Fig. 4, Table 1). The tumor movements (mean ± SD) from prone to supine at the nipple origin and at the origin of the bottom center of the sternum were 27.8 ± 3.3 and 63.1 ± 26.2 mm, respectively (Table 1). The tumor movement from prone to supine positions at the origin of the bottom center of the sternum was greater than those at the nipple origin. Supplementary Fig. S1 shows coronal (X-Z) and axial (X-Y) views of the bilateral breasts after fixing the nipple origin (Table 2) and Fig. 5 shows a case of tumor movement at the nipple origin from prone to supine positions in frontal and right-side view of 3D body surface. Figure 6 shows the coronal, sagittal, and axial views of the bilateral breasts at the origin of the bottom center of the sternum (0, 0). Table 3 presents the prone-to-supine movement data for the 27 patients. Ten patients (37%) had outward and upward movements, while the remaining 17 patients (63%) had outward and downward movements from prone to supine positions. In Fig. 7, tumor movement from prone to supine position at the origin of the bottom center of the sternum was strongly correlated with the distance from the tumor center to the chest wall (*r* = 0.669; *p* < 0.05). However, there is no significant correlation at the origin.

**Table 1:** Tumor volume changes at each position and tumor movement from prone to supine positions.

Position	Prone	Supine
Volume change $({{\rm{mm}}}^{3})$	9,797.3 ± 9,390.6	9,795.5 ± 9,413.1
**Origin**	**Nipple**	**Bottom center of the sternum**
Total movement (${\rm{mm}}$)	27.8 ± 3.3	63.1 ± 26.2

**Table 2:** Prone-to-supine movement at the nipple origin in coronal (X-Z) and axial (X-Y) views.

Coronal view		Axial view	
Outward and upward	Outward and downward	Outward and downward	Outward and upward
11/13 (84.6%)	2/13 (15.4%)	20/27 (74.1%)	7/27 (25.9%)

**Table 3:** Prone-to-supine movement at the origin of the bottom center of the sternum in coronal (X-Z) and sagittal (Y-Z) views.

Coronal view		Sagittal view	
Outward and upward	Outward and downward	Inward and upward	Inward and downward
17/27 (63%)	10/27 (37%)	17/27 (63%)	10/27 (37%)

## Discussion and Conclusions

To improve diagnostic accuracy, MRI is performed at the prone position with a special breast coil. However, the surgical position is a supine, which is different from the position of the tumor observed in the prone position during an MRI scan. Although MRI is known to have high accuracy in predicting the extent of tumors, the supine posture during surgery differs from the prone posture during an examination, and the position of the tumor may change due to posture changes. Thus, inaccuracy in the identification of tumor position can occur in surgery planning. The results of this study could be helpful to surgeons during breast resection by predicting the change of tumor position according to posture changes.

To determine the exact surgical area using MRI, the volume change and prone-to-supine movement of breast tumors pre-operation were evaluated. No difference in tumor volume according to posture change was found. This differs from a study by Gombos *et al*.^15^ which observed an average change of 23.8% in the measured tumor volume in 12 patients. This is likely due to the fact that this study only had six cases that underwent both pre- and post-surgical supine MRI. Samani *et al*.^16^, however, showed that fibrocystic disease and malignant tumors exhibited a three- to six-fold increase in stiffness, with high-grade invasive ductal carcinoma exhibiting up to a 13-fold increase in stiffness compared to fibrous tissue. There is a difference in breast density for Asian and Western women^17^, which can produce different results.

The data presented in Table 1 indicates that there was little volume change in the prone position and supine position and that the tumor movement from prone to supine positions at the origin of the bottom center of the sternum was greater than that at the nipple origin. The distance from the tumor center to the chest wall is measured by the depth of the tumor in the breast, which could be an important factor in predicting tumor movement. However, among the possible landmarks in the ribs and sternum, the bottom center of the sternum was determined as the origin as it is easy to find.

Supplementary Fig. S1 and Table 2 shows that from the viewpoint of the nipple origin, prone-to-supine tumor movement in the breast is variable depending on tumor location. The vectors of the 27 patients’ movements from the prone to supine positions are on the coronal and axial planes, respectively. Tumor locations were plotted on each coordinate, and the vectors for prone-to-supine tumor movements were drawn by connecting the two points, including the tumor centers in the prone and supine positions of each tumor. If the movement of a tumor is |X| > |X’|, it is outward, and if it is |X| < |X’| it is inward in coronal view. If the movement is Z < Z’, it is upward, and if it is Z > Z’ it is downward in coronal view (Supplementary Fig. S1A). Supplementary Fig. S1B shows that if the movement of the tumor is |X| > |X’|, it is outward and if it is |X| > |X’|, it is inward in axial view. If the movement is Y > Y’, it is upward and when it is Y < Y’ it is downward in axial view. By changing the patient’s position from prone to supine, tumors located in the outer area tended to move outward and downward along the z-axis (Fig. 5). Otherwise, the directions of tumor movement in the inner area (where the tumor’s X coordinate is greater than 0 in coronal view) were irregular. Taking into account changes in the axial plane from the prone to supine position shown in Supplementary Fig. S1B, breast tumors tended to move outward and downward along the axial view in the entire breast area. The tumor movements of the 27 patients in the sagittal view were irregular. This result suggests that the location of the tumor in the supine position could be predicted when the tumors are located in the outer area in prone MRI.

The inconsistency of movement can be attributed to the very large movement of the nipple, which was regarded as the origin. Tumor movement from the origin of the bottom center of the sternum (0, 0) was relatively robust to posture and respiratory changes. Tumor locations in prone and supine positions were plotted on each coordinate, and tumor movement vectors from prone to supine positions were drawn by connecting the two points of each tumor’s center. As above, |X| > |X’| indicates outward movement and |X| < |X’| indicates inward movement in coronal view, while Z < Z’ indicates upward movement and Z > Z’ indicates downward movement in coronal view (Fig. 6A). If the movement is Y<Y’ this indicates outward movement and if it is Y > Y’ it indicates inward movement in sagittal view (Fig. 6B). Figure 6C shows that if tumor movement is Z < Z’, it is upward and if it is Z > Z’ it is downward in axial view. As shown in Fig. 6A and Table 3, all breast tumors tended to move outward from the centerline of the body on a coronal plane and move toward the body on the sagittal and axial planes. For the prone-to-supine tumor movement on the axial plane (Fig. 6C), all breast tumors tended to move outward and closer to the chest wall. All cases displayed outward and inward prone-to-supine movement.

During breast MRI scanning in the prone position, the breasts would be pendant and uncompressed, and tumors might appear to be located more anteriorly and closer to the nipple position. In the operating room, the patient lays in a supine or supine oblique position, with the corresponding arm raised behind the head or neck to reduce breast thickness and movement. All tissue layers are flattened and widened, especially the fatty tissue, which is relatively more highly compressible and stretchable than other breast structures. Due to the stiffness of the tumor, its deformation is limited. In addition, position in the breast in the supine position could be changed due to the interaction between gravity and the curvature of the chest wall. We found that if a tumor is located closer to the breast skin, the deformation and movements might be affected by the tension of the skin. In this way, considering the lesion’s characteristics and surrounding tissues, it is helpful to understand the deformation of the tumor according to posture change.

This study was subject to several limitations. First, we studied a small number of breast cancer patients (27 patients with 43 breast cancer tumors). However, each tumor consisted of a prone and supine MRI pair showing pre-NST and post-NST, which were repeatedly measured in order to evaluate volume change and tumor movement more robustly. Further studies with a larger patient population are required. Moreover, owing to the use of delayed mDixon imaging in supine MRI and their washing out of contrast media, small tumors or ductal carcinoma *in situ* (DCIS) could not be detected. In the case of a tumor with a paradoxical volume change (increased volume in the supine position; Fig. 4), the type of breast cancer observed in this patient was metaplastic carcinoma with osseous differentiation, which was not as common in the other patients. These pathological features may have different effects on volume changes, but further study is necessary. In the future, factors such as the distance from the lesion to the nipple origin or the skin, the size of the tumor, the stiffness of the tumor, the volume of the breast, the patient’s age, and menopausal status may affect the movement of the tumor in more cases. According to our results, it may be possible to predict the movement of a tumor according to the posture more accurately. Nevertheless, this is the first report that directly compares tumor movement in 3D coordinates using prone and supine MRIs. For further studies, these factors need to be considered. In addition, using both prone and supine MRIs has the potential to evaluate patients more accurately and quantitatively and could be used in various applications including surgical and radiotherapy planning, image guided interventions; and multi-modal tumor diagnosis, staging, and therapy response prediction.

In conclusion, tumors located in the outer position of the breast in prone position tend to move outward and downward from the sagittal centerline of the breast in supine position. The average movement of the tumor between prone and supine positions was no more than 30 mm using the nipple origin. The origin of the bottom center of the sternum could be regarded as a better origin. In addition, tumor movement from the origin of the bottom center of the sternum was strongly correlated with the distance from the tumor center to the chest wall.

## Supplementary information

Supplementary information.
