# Supplementary material for: Breast tumor movements analysis using MRI scans in prone and supine positions
Source: Sci Rep. 2020 Mar 17;10:4858. doi: 10.1038/s41598-020-61802-9 (PMC7078262; doi:10.1038/s41598-020-61802-9)
Supplement: Supplementary file 1 — Supplementary information. [file 41598_2020_61802_MOESM1_ESM.pdf]

Breast tumor movements analysis using MRI scans in prone and supine positions

Chuan-Bing Wang[1,2,a], Sangwook Lee[2,3,a], Taehun Kim[2,3], Dayeong Hong[2,3], Guk Bae Kim[4], Ga Young Yoon[2], Hak Hee Kim[2], Namkug Kim[2,3,*], BeomSeok Ko[5,*]

[1] *Department of Radiology, First Affiliated Hospital of Nanjing Medical University, 300 Guangzhou Road, Nanjing, Jiangsu, China.*

[2]*Department of Radiology, University of Ulsan College of Medicine, Asan Medical Center, 388-1 Pungnap2-dong, Songpa-gu, Seoul, South Korea*

[3]*Department of Convergence Medicine, University of Ulsan College of Medicine, Asan Medical Center, 388-1 Pungnap-dong, Songpa-gu, Seoul, South Korea*

[4]*ANYMEDI Inc., 388-1 Pungnap-dong, Songpa-gu, Seoul, South Korea*

[5]*Department of Breast Surgery, University of Ulsan College of Medicine, Asan Medical Center, 388-1 Pungnap-dong, Songpa-gu, Seoul, South Korea*

[a]These two authors contributed equally as the first authors.

*These two authors contributed equally as the corresponding authors.

Supplementary Fig. S1 Coronal (X-Z) and axial (X-Y) views of the bilateral breasts at the nipple origin. The vectors of the 27 patients' movements from the prone to supine positions in the coronal view (A) and the axial view (B) are shown (unit: mm).

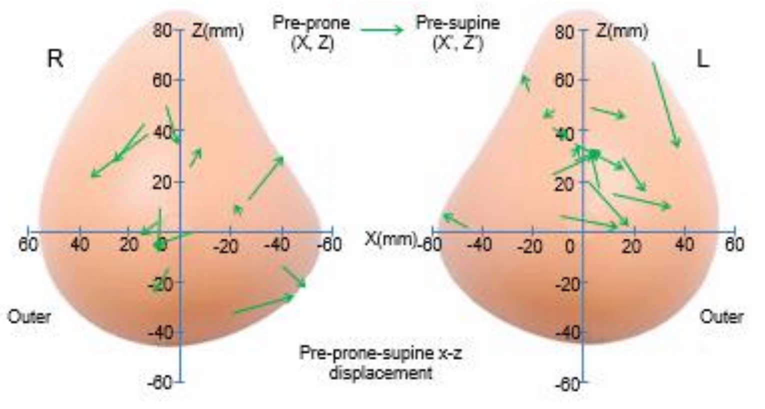

A

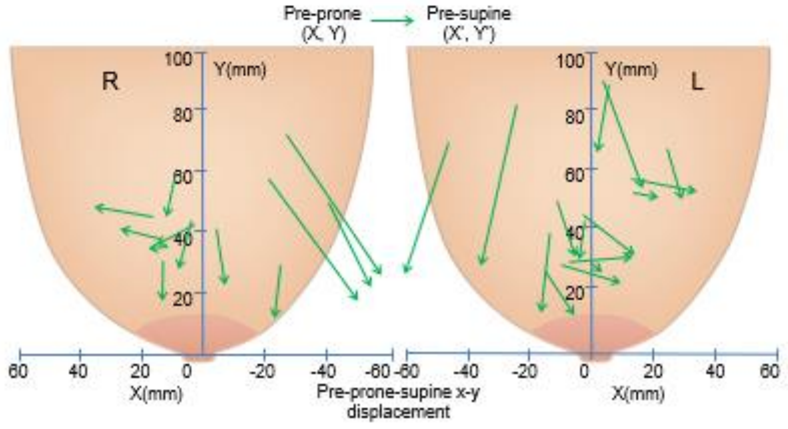

B
